# High-fat Western diet consumption exacerbates silica-induced pulmonary inflammation and fibrosis

**DOI:** 10.1016/j.toxrep.2022.04.028

**Published:** 2022-05-02

**Authors:** Janet A. Thompson, Richard A. Johnston, Roger E. Price, Ann F. Hubbs, Michael L. Kashon, Walter McKinney, Jeffrey S. Fedan

**Affiliations:** a Health Effects Laboratory Division, National Institute for Occupational Safety and Health, Morgantown, WV 26508, United States; b Veterinary Pathology Services, Magnolia, TX, 77354, United States

**Keywords:** Silicosis, Metabolic syndrome, High-fat diet, Inflammation, Lung

## Abstract

Consumption of a high-fat Western diet (HFWD) contributes to obesity, disrupted adipose endocrine function, and development of metabolic dysfunction (MetDys). Impaired lung function, pulmonary hypertension, and asthma are all associated with MetDys. Over 35% of adults in the U.S. have MetDys, yet interactions between MetDys and hazardous occupational inhalation exposures are largely unknown. Occupational silica-inhalation leads to chronic lung inflammation, progressive fibrosis, and significant respiratory morbidity and mortality. In this study, we aim to determine the potential of HFWD-consumption to alter silica-induced inflammatory responses in the lung. Six-wk old male F344 rats fed a high fat Western diet (HFWD; 45 kcal % fat, sucrose 22.2% by weight) to induce MetDys, or standard rat chow (STD, controls) for 16 wk were subsequently exposed to silica (6 h/d, 5 d/wk, 39 d; Min-U-Sil 5®, 15 mg/m^3^) or filtered air; animals remained on their assigned diet for the study duration. Indices of lung inflammation and histopathologic assessment of lung tissue were quantified at 0, 4, and 8 wk after cessation of exposure. Combined HFWD+silica exposure increased bronchoalveolar lavage (BAL) total cells, leukocytes, and BAL lactate dehydrogenase compared to STD+silica exposure controls at all timepoints. HFWD+silica exposure increased BAL proinflammatory cytokines at 4 and 8 wk compared to STD+silica exposure. At 8 wk, histopathological analysis confirmed that alveolitis, epithelial cell hypertrophy and hyperplasia, lipoproteinosis, fibrosis, bronchoalveolar lymphoid hyperplasia and granulomas were exacerbated in the HFWD+silica-exposed group compared to STD+silica-exposed controls. Our results suggest an increased susceptibility to silica-induced lung disease caused by HFWD consumption.

## Highlights

• HFWD exacerbates silica (SIL)-induced lung injury at 8 wk post-exposure.
• HFWD+SIL increases BAL cells and LDH compared to STD+SIL.
• HFWD+SIL increases BAL proinflammatory cytokines compared to STD+SIL.
• Histopathology confirms exacerbated lung injury HFWD+silica treatment.

## Graphical Abstract

**Figure:**
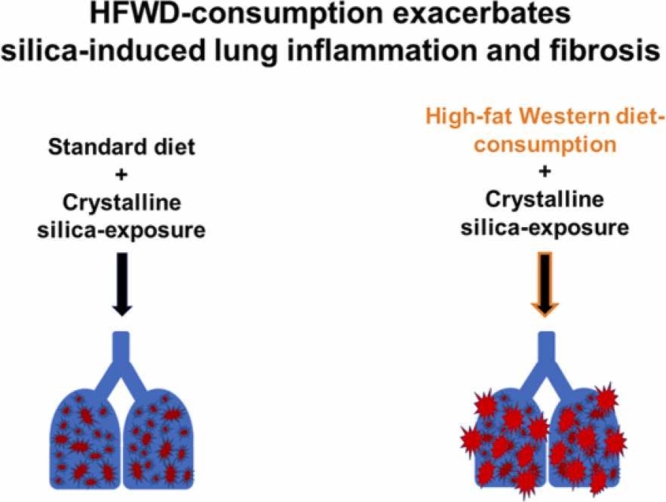

## 1 Introduction

Consumption of a high-fat Western diet (HFWD) contributes to obesity, disruption of normal adipose endocrine function, and development of metabolic dysfunction (MetDys). Over thirty-five percent of adults in the U.S. have metabolic dysfunction (MetDys) [1]. MetDys risk factors include abdominal obesity, elevated triglycerides, decreased high-density lipoprotein (HDL), insulin resistance and elevated blood glucose, hypertension, inflammation, oxidative stress, and a prothrombotic state [2]. In addition, MetDys is associated with many disease states including type 2 diabetes, cardiovascular disease, stroke, non-alcoholic fatty liver disease, polycystic ovary syndrome, neurological disorders, and cancers [2]. Obesity is central to development of MetDys, which originates with obesity-induced adipose tissue dysfunction, resulting in altered serum levels of adipocytes leptin and adiponectin and induction of chronic systemic inflammation [3].

MetDys may influence a worker’s susceptibility to occupational inhalation hazards. The presence of pre-existing MetDys biomarkers (elevated triglycerides, heart rate and leptin and low HDL) were associated with increased susceptibility to severe lung function impairment in first responders following the 9/11 World Trade Center attack [4], [5], [6], [7]. Silicosis cases in Appalachian coal miners increased in number between 1970 and 2015 [8], [9], while participants of the 2015 Enhanced Coal Workers' Health Surveillance Program (ECWHSP), which included coal miners from Appalachia, had a higher rate of being overweight (87%), obese (52%), or having hypertension (31%), compared to the general population [10]. Another study found that coal miners diagnosed with silicosis and MetDys were at increased risk for atherosclerosis [10]. Further associations between diet and occupational-exposure related toxicity have been shown in animal studies. Rats fed a HFWD and exposed to occupational welding fumes developed increased kidney toxicity, altered serum enzyme and protein levels [11], [12], and the hepatic lipidome was altered [12], compared to animals fed a standard diet (STD).

Silicosis is an irreversible progressive lung disease caused by occupational exposure and inhalation of respirable crystalline silica dust [13]. The pathogenesis of silicosis begins with deposition of inhaled silica particles into the alveolar regions of the lung where they encounter and become phagocytosed by alveolar macrophages (AMs) [14]. Phagocytosed silica is transported intracellularly to the lysosome, where it interacts with and disrupts the lysosomal membrane. Leaking of lysosomal contents into the cytoplasm leads to activation of the AM NLRP3 inflammasome [15], AM apoptosis or necrosis, and, ultimately, the release of caustic AM contents and silica particles back into the alveolar space to be phagocytosed by other AMs in a positive feedback loop. Activated AMs release free radicals, inflammatory cytokines, leukotrienes, proteases, chemokines and chemoattractant proteins, reactive oxygen species (ROS), and reactive nitrogen species (RNS) into the alveolar space [16], [17], [18], [19], [20]. Cytokines, including TNF-α, IL-1β and TGF-β, are released by AMs and activate alveolar fibroblasts to deposit collagen and elastin, and contribute to the development of pulmonary fibrosis. This cycle of release and reuptake of silica particles, AM apoptosis, pulmonary inflammation, and fibrosis, all contribute to the progression of silicosis [21], [22], [23], [24]. Previously, our laboratory demonstrated in a rat model that inhalation of crystalline silica markedly altered the profile of many metabolic and inflammatory changes caused by a HFWD [25], i.e., silica-inhalation altered adipose function in animals with pre-existing HFWD-induced MetDys. Surprisingly, serum adipokines, leptin and adiponectin, as well as serum proinflammatory cytokines levels, were reduced at 8 wk after exposure to crystalline silica inhalation in animals that consumed a HFWD when compared to STD fed controls.

This hazard identification study is part of an ongoing investigation aimed at investigating the effects of combined HFWD-consumption and inhalation exposure to respirable crystalline silica. In this study, we test the hypothesis that HFWD-consumption increases silica-inhalation induced lung injury and inflammation in the Fischer F344 rat model. We also characterize the interactions of diet and silica exposure effects on lung histopathology. To our knowledge, there are no previous studies investigating the potential interactions of HFWD-consumption and silica inhalation in the context of pulmonary injury and inflammation.

## 2 Materials and methods

### 2.1 Animals and diet

All studies were conducted in facilities accredited by AAALAC International, were approved by the CDC-Morgantown Institutional Animal Care and Use Committee. All animals were free of viral pathogens, parasites, mycoplasmas, *Heliobacter* and cilia-associated respiratory bacillus. Animals were acclimated for one week upon arrival and housed in ventilated micro-isolator units supplied with HEPA-filtered laminar flow air (Lab Products OneCage; Seaford, DE), with Teklad Sanichip and Teklad Diamond Dry cellulose bedding (or Shepherd Specialty Paper’s Alpha-Dri cellulose; Shepherd Specialty Papers; Watertown, TN). Animals were provided filtered tap water and housed in pairs under controlled light cycle (12 h light/12 h dark) and temperature (22 – 25 °C) conditions.

**Fig. 1:**
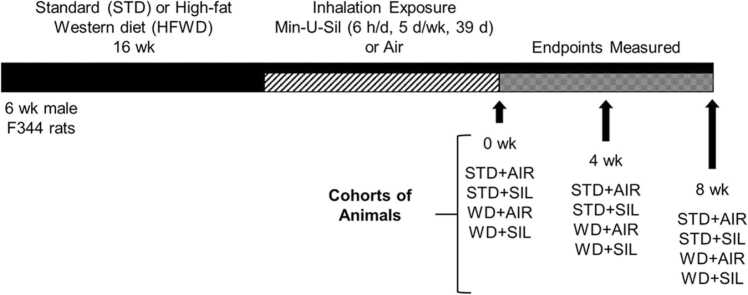
Experimental design for HFWD-induction of MetDys, silica inhalation exposure, and endpoint measurements, using separate cohorts of animals (n = 8 for each group).

Reproduced from Thompson et al. [25] with modifications.

Six-wk old male Fischer (CDF) rats (F344/DuCrl) obtained from Charles River Laboratories Inc. (Wilmington, MA) were divided into two dietary groups and fed either a commercially available “Western” diet (HFWD; Teklad TD.06415 Western diet; Envigo, Indianapolis, IN) containing a nutritional profile of: 45% fat Kcal; sucrose 22.2% by weight; fatty acid profile of 35% saturated, 40% monounsaturated, 25% polyunsaturated; or a standard rat chow (standard diet, STD; fat 6.2% by weight, sucrose-free) for 16 wk, prior to the commencement of silica dust inhalation exposures. After 16 wk, animals were exposed to silica dust for 6 h/d, 5 d/wk, 39 d or filtered air (control) and handled identically. Endpoint measures were obtained at 0, 4, and 8 wk post-exposure (Fig. 1).

### 2.2 Silica exposure

Crystalline silica (Min-U-Sil 5®; Berkeley Springs, WV; “SIL”) was aerosolized using a custom automated exposure system described previously [26] that delivered airborne particles with median aerodynamic diameter of 1.6 µm and geometric standard deviation of 1.6. Target silica concentration (15 ± 1 mg/m^3^) was monitored and controlled within the exposure chamber in real time; control animals were exposed to filtered air and handled identically.

### 2.3 Bronchoalveolar Lavage (BAL) and Lung Histology

Animals were euthanized by an overdose of sodium pentobarbital (200–300 mg/kg, Vortech; Dearborn, Michigan) administered by intraperitoneal injection. With the left lung clamped, the right lung was used for collection of BAL fluid. After exposing the trachea, a small incision was made, and a catheter was inserted into the lumen and secured with suture. The right lung was lavaged five times with 5 ml of ice-cold PBS. The first lavage was kept separate from the four subsequent washes and all washes were stored on ice until further use; BAL samples were then centrifuged at 600*g* for 10 min. The supernatant from the first lavage was removed, aliquoted, and used for lactate dehydrogenase (LDH) analysis; all remaining supernatant aliquots were stored at − 80 °C for cytokine analysis.

The cell pellet from the first lavage was resuspended in 1 ml of PBS and combined with the cell pellet from the subsequent lavage sample. First, the total number of BAL cells was determined using electronic cell counter/sizer (Coulter Multisizer II, Coulter Electronics; Hialeah, FL). The remaining BAL cells were spun onto a glass microscope slide using a Cytospin 3 Cytocentrifuge (Thermo Shandon; Pittsburgh, PA). Slides were air-dried and then stained with Hema 3 (Biochemical Sciences; Swedesboro, NJ). Three hundred cells were counted under light microscopy by a board-certified veterinary pathologist (AFH) for the differential cell analysis.

### 2.4 BAL cytokine analysis

BAL cytokines (Table 1) were measured using the MSD V-PLEX Proinflammatory Panel 2 (rat) kit and MESO QuickPlex SQ 120 (Meso Scale Diagnostics; Rockville, MD) following the manufacturer’s protocol.

**Table 1:** BAL proinflammatory cytokines measured.

Cytokine	Abbreviation	Other names
Interferon gamma	IFN-γ	Type II interferon
Interleukin-1β	IL-1β	IL-1F2
Interleukin-4	IL-4	B-Cell stimulatory factor 1 (BSF-1); lymphocyte stimulatory factor 1
Interleukin-5	IL-5	B-cell growth factor II (BCGF-II); T-cell replacing factor (TRF); eosinophil differentiation factor (EDF)
Interleukin-6	IL-6	
Keratinocyte chemoattractant	KC/GRO	CXCL1; CINC-1; GRO-α, neutrophil-activating protein 3 (NAP-3); melanoma growth stimulating activity α (MGSA-α)
Tumor necrosis factor-α	TNF-α	Tumor necrosis factor ligand superfamily member 2 (TNFSF2); cachectin

### 2.5 Lung histology

Following lavage of the right lung, the right lung was tied off and removed. The left lung lobe, which had not been lavaged, was inflated with 3.5 ml of 10% neutral buffered formalin and collected for histological examination. The left lung was paraffin-embedded and 5 µm sections were prepared for hematoxylin and eosin (H&E), Masson’s trichrome, and picrosirius red staining in order to examine sections for indicators of pulmonary injury, inflammation, and fibrosis by a board-certified veterinary pathologist (R.E.P.). An informed unblinded, semi-quantitative histopathologic assessment was used, as recommended by the Society of Pathology [27] and the National Toxicology Program [28], to ascertain the distribution and severity of histopathological changes in the lungs at 0, 4 and 8 wk post-exposure in the four animal cohorts.

### 2.6 Statistical analysis

Data were analyzed using JMP version 13.2, and SAS version 9.4 (SAS Institute; Cary NC). Variables were analyzed using three-way analyses of variance (diet × treatment × time). Relevant pair-wise comparisons were generated using Fisher’s LSD test. For cytokine analysis, all values below the lower limit of detection (LLOD) were replaced with the LLOD/sqrt(2). Pathology scores were analyzed using the nonparametric Kruskal-Wallis test followed by the Wilcoxon Rank-sum test for pairwise comparisons. All differences were considered significant at P < 0.05.

## 3 Results

**Fig. 2:**
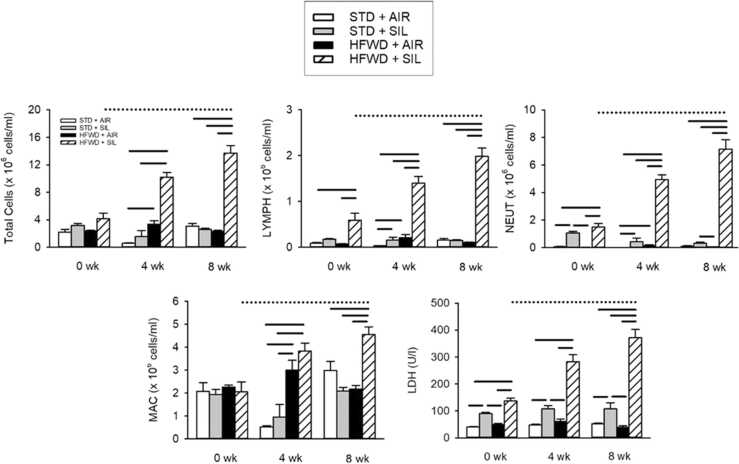
Effects of silica inhalation, HFWD and combined HFWD+SIL exposure on BAL total cells, cell differentials and LDH. Total cells (TC), lymphocytes (LYMPH), neutrophils (NEUT), macrophages (MAC) and lactate dehydrogenase (LDH) were measured in BAL fluid. Silica exposure increased NEUT at 4 and 8 wk, while LDH levels were increased at all timepoints, compared to the STD+AIR control. HFWD+AIR increased number of TC, LYMPH and MAC at 4 wk compared to STD+AIR. HFWD+SIL increased the number of BAL TC, LYMPH, NEUT, MAC and LDH at 4 and 8 wk, compared to STD+SIL animals. Solid lines indicate significant differences between different treatment groups at a given time point. Dotted lines indicate significant differences vs. time within the HFWD+SIL group. (P < 0.05, n = 7–8).

BAL LDH, total and cell differentials were measured at 0, 4, and 8 wk post-exposure to silica in both HFWD and STD groups (Fig. 2). Silica exposure significantly increased the numbers of BAL lymphocytes (LYMPH) and neutrophils (NEUT) at 4 wk compared to the STD+AIR group, and neutrophils at 8 wk compared to the HFWD+AIR group. Silica inhalation increased BAL LDH at all timepoints compared air-exposed groups regardless of diet. Consumption of the HFWD alone increased the levels of total cells, LYMPH, NEUT and macrophages (MAC) at 4 wk compared to STD+AIR; at 8 wk there were no longer any differences. HFWD alone had no effect on BAL LDH at any timepoint. Combined exposure HFWD+SIL had profound effects at 4 and 8 wk, with significant increases occurring in BAL TC, LYMPH, NEUT, and MAC in comparison to the other exposure groups. HFWD+SIL increased LDH levels to a greater degree than that in other groups at all timepoints.

**Fig. 3:**
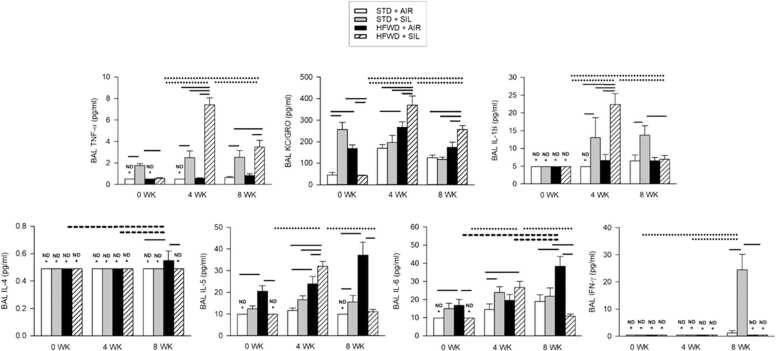
Effects of HFWD and silica inhalation on BAL pro-inflammatory cytokines. Silica inhalation had varied effects on inflammatory cytokine levels compared to the STD+AIR controls. Solid lines indicate significant differences between different treatment groups at a given time point. Dashed lines indicate significant difference vs. time within the HFWD+AIR group. Dotted lines indicate significant differences vs. time within the HFWD+SIL group. ND indicates cytokine levels below LLOD (pg/ml): IFN-γ = 0.65; IL-1β = 6.92; IL-4 = 0.69; IL-5 = 14.10; IL-6 = 13.80; KC/GRO = 1.04; TNF-α = 0.72. P < 0.05. n = 7–8.

Pulmonary inflammation is induced in response to crystalline silica inhalation, and, therefore, proinflammatory cytokines were measured in the BAL fluid at 0, 4 and 8 wk after silica exposure (Fig. 3). Silica had a variety of effects on the levels of the different cytokines. TNF-α levels were increased and sustained at all timepoints, while KC/GRO increased at 0 wk, IL-1β increased at 4 and 8 wk, IL-6 increased at 4 wk, IL-5 and INF-γ were increased at 8 wk, compared to the STD+AIR controls. Consumption of the HFWD increased IL-5 levels at all time points, KC/GRO at 4 wk, and IL-4 and IL-6 at 8 wk, compared to the STD+AIR controls. At 8 wk, IL-4 and IL-6 levels were greater compared to those measured at earlier timepoints within the HFWD group. The combined HFWD+SIL exposure resulted in significant increases in the levels of TNF-α, KC/GRO, IL-1β, IL-5, compared to all other groups at 4 wk; however, of great interest was the peak response of these cytokines in BAL at 4 wk vs. the other timepoints within the HFWD+SIL group, and the sustained increase in BAL KC/GRO at 8 wk compared to all other groups at that timepoint. We also found in the HFWD+SIL groups that TNF-α and KC/GRO levels were reduced at 0 wk, and IL-1β and IL-6 were decreased at 8 wk compared to the STD+SIL cohort.

**Fig. 4:**
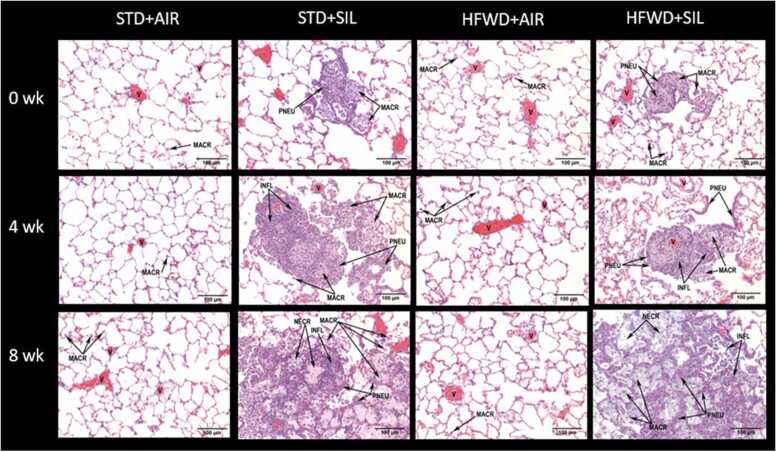
Effects of consumption of HFWD on silica-induced pulmonary inflammation. Silica inhalation resulted in pulmonary inflammation which increased in both distribution and severity over time. Abbreviations: MACR: macrophage; PNEU: pneumocyte; NECR: necrosis/cellular detritus; INFL: mononuclear/mixed inflammatory cell infiltrates; V: blood vessel; COLL: collagen. HFWD+SIL exposure produced lesions like those following STD+SIL exposure. At 8 wk, there were greater levels of necrosis/cellular detritus, mononuclear/mixed inflammatory cell infiltrates, macrophages, and pneumocytes in the HFWD+SIL lesions compared to STD+SIL-treated animals. Images were stained with H&E. Bar equals 100 µm. (P < 0.05, n = 7–8).

**Fig. 5:**
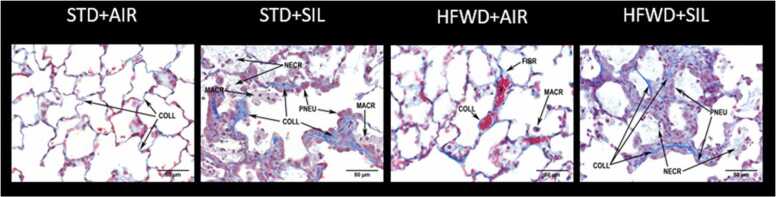
Effects of consumption of HFWD on silica-induced fibrosis at 0, 4 and 8 wk. Collagen deposition from animals exposed to silica dust and/or fed HFWD was examined using Mason’s trichrome stain and examined for the presence of fibrosis. Fibrosis was observed in both STD+SIL and HFWD+SIL-exposed groups at 8 wk. Fibrosis was more severe in the HFWD+SIL groups compared to STD+SIL. Images stained with Mason’s trichrome are shown at 40x magnification. Abbreviations: COLL: collagen; MACR: macrophage; PNEU: pneumocyte; NECR: necrosis/cellular detritus. Bar equals 100 µm. P < 0.05. n = 7 – 8.

Semiquantitative histopathologic assessment confirmed that silica dust induced lung injury at all three timepoints measured, regardless of diet group (Table 2, Table 3). At 0 wk, silica induced multi-focal lesions which consisted of aggregates of plump alveolar macrophages along with alveolar epithelial (pneumocyte) hypertrophy occurring with minimal severity (<10% area) in both diet groups (Fig. 4). Lesions at 8 wk were characterized by the presence of accumulation of infiltrated cells, necrotic detritus, increased deposition of collagen (Fig. 5), and emphysematous-like structures in the alveolar space. At 8 wk, alveolar lesions (both alveolitis and epithelial hyperplasia) distribution increased to multifocal and coalescent in both diet groups. While the lesion severity of the STD+SIL group increased from minimal to mild (10–25% area), the lesion severity of the HFWD+SIL group was even greater increasing from minimal to moderate (26–50% area), indicating lung injury greater than the STD+SIL control group at 8 wk. Lipoproteinosis, a characteristic feature of the acute response to silica inhalation in humans [40] and a response that accompanies alveolar epithelial hypertrophy and hyperplasia in silica-exposed rats [18], [39], [40], was present at 8 wk in both diet groups; however, severity was greater in the HFWD+SIL group (moderate severity, 26–50% area) compared to the STD+SIL group (mild severity, 10–25% area). Fibrosis was present at 8 wk in both diet groups but was more prevalent and more severe in the HFWD+SIL group (moderate, 26–50% area) compared to the STD+SIL group (mild,10–25% area). In the bronchus-associated lymphoid tissue (BALT) region, granulomatous lesions and cell hyperplasia were present at all timepoints and were not different between the diet groups nor significantly different over time between 0 and 8 wk within any group.

**Table 2:** Semiquantitative histopathologic assessment of alveolitis, epithelial cell hypertrophy and hyperplasia, lipoproteinosis, and fibrosis.

	Alveolar								Bronchus-associated lymphoid tissue (BALT)			
	Alveolitis		Epithelial Cell Hypertrophy and Hyperplasia		Lipoproteinosis		Fibrosis		Granulomatous Inflammation		Hyperplasia	
	Distribution	Severity	Distribution	Severity	Distribution	Severity	Distribution	Severity	Distribution	Severity	Distribution	Severity
STD+AIR 0 wk	0.0	0.0	0.0	0.0	0.0	0.0	0.0	0.0	0.0	0.0	0.0	0.0
HFWD+AIR 0 wk	0.0	0.0	0.0	0.0	0.0	0.0	0.0	0.0	0.0	0.0	0.0	0.0
STD+SIL 0 wk	3.0	1.0	3.0	1.0	0.0	0.0	0.0	0.0	2.5 ± 0.3	1.1 ± 0.1	3.8 ± 0.8	0.9 ± 0.2
HFWD+SIL 0 wk	3.0	1.0	3.0	1.0	0.0	0.0	0.0	0.0	1.6 ± 0.6	1.1 ± 0.4	1.9 ± 0.9	0.9 ± 1.05
												
STD+AIR 4 wk	0.0	0.0	0.0	0.0	0.0	0.0	0.0	0.0	0.0	0.0	0.0	0.0
HFWD+AIR 4 wk	0.0	0.0	0.0	0.0	0.0^**#**^	0.0^**#**^	0.0	0.0	0.0	0.0	0.0	0.0
STD+SIL 4 wk	3.0	1.0	3.0	1.0	0.0	0.0	0.0	0.0	2.9 ± 0.4	2.6 ± 0.4	4.4 ± 0.6	2.6 ± 0.4
HFWD+SIL 4 wk	3.0	1.0	3.0	1.0	0.0	0.0	0.0	0.0	1.8 ± 0.5	1.1 ± 0.3	2.5 ± 1.0	1 ± 0.4
												
STD+AIR 8 wk	0.0	0.0	0.0	0.0	0.0	0.0	0.0	0.0	0.0	0.0	0.0	0.0
HFWD+AIR 8 wk	0.0	0.0	0.0	0.0	0.0	0.0	0.0	0.0	0.0	0.0	0.0	0.0
STD+SIL 8 wk	4.0	2.0	4.0	2.0	4.0	2.0	4.0	1.0	2.6 ± 0.4	1.5 ± 0.3	4.4 ± 0.6	1.4 ± 0.3
HFWD+SIL 8 wk	4.0	3.0*	4.0	3.0*	4.0	3.0*	4.0	2.0*	3.0	2.0 ± 0.3	3.8 ± 0.8	1.6 ± 0.4

Semi-quantitative histopathologic assessment was used to determine distribution and severity of silica-induced lesions in the lung at 0, 4 and 8 wk post-exposure to silica. Distribution: 0 = none, 1 = focal, 2 = locally extensive, 3 = multifocal, 4 = multifocal and coalescent, and 5 = diffuse. Severity/area: 0 = none, 1 = minimal (<10%), 2 = mild (10–25%), 3 = moderate (26–50%), 4 = marked (51–75%), and 5 = severe (76–100%). ^#^ n = 1 scored lipoproteinosis. *Significant difference compared to STD+SIL 8 wk. P < 0.05. n = 8.

**Table 3:** Summary of histopathological alterations.

Exposure Group	Alveolar								(BALT)			
	Alveolitis		Epithelial Cell Hypertrophy and Hyperplasia		Lipoproteinosis		Fibrosis		Granulomatous Inflammation		Hyperplasia	
	Distribution	Severity	Distribution	Severity	Distribution	Severity	Distribution	Severity	Distribution	Severity	Distribution	Severity
STD+AIR 0 wk	0.0 (8)	0.0 (8)	0.0 (8)	0.0 (8)	0.0 (8)	0.0 (8)	0.0 (8)	0.0 (8)	0.0 (8)	0.0 (8)	0.0 (8)	0.0 (8)
HFWD+AIR 0 wk	0.0 (8)	0.0 (8)	0.0 (8)	0.0 (8)	0.0 (8)	0.0 (8)	0.0 (8)	0.0 (8)	0.0 (8)	0.0 (8)	0.0 (8)	0.0 (8)
STD+SIL 0 wk	3.0 (8)	1.0 (8)	3.0 (8)	1.0 (8)	0.0 (8)	0.0 (8)	0.0 (8)	0.0 (8)	1.0 (2) 3.0 (6)	1.0 (7) 2.0 (1)	0.0 (2) 5.0 (6)	0.0 (2) 1.0 (5) 2.0 (1)
HFWD+SIL 0 wk	3.0 (8)	1.0 (8)	3.0 (8)	1.0 (8)	0.0 (8)	0.0 (8)	0.0 (8)	0.0 (8)	0.0 (3) 1. 0 (2) 3. 0 (1) 4. 0 (2)	0.0 (3) 1. 0 (3) 3. 0 (2)	0. 0 (5) 5. 0 (3)	0.0 (5) 1. 0 (1) 3. 0 (2)
												
STD+AIR 4 wk	0.0 (8)	0.0 (8)	0.0 (8)	0.0 (8)	0.0 (8)	0.0 (8)	0.0 (8)	0.0 (8)	0.0 (8)	0.0 (8)	0.0 (8)	0.0 (8)
HFWD+AIR 4 wk	0.0 (8)	0.0 (8)	0.0 (8)	0.0 (8)	0.0 (7) 1.0 (1)	0.0 (7) 1.0 (1)	0.0 (8)	0.0 (8)	0.0 (8)	0.0 (8)	0.0 (8)	0.0 (8)
STD+SIL 4 wk	3.0 (8)	1.0 (8)	3.0 (8)	1.0 (8)	0.0 (8)	0.0 (8)	0.0 (8)	0.0 (8)	0.0 (1) 3. 0 (5) 4. 0 (2)	0.0 (1) 3.0 (7)	0.0 (1) 5. 0 (7)	0.0 (4) 2. 0 (4)
HFWD+SIL 4 wk	3.0 (8)	1.0(8)	3.0 (8)	1.0 (8)	0.0 (8)	0.0 (8)	0.0 (8)	0.0 (8)	0.0 (2) 1. 0 (2) 3. 0 (4)	0.0 (2) 1. 0 (2) 3. 0 (1) 4. 0 (2)	0.0 (3) 1. 0 (3) 2. 0 (3)	0.0 (3) 1. 0 (2) 3. 0 (1) 4. 0 (2)
												
STD+AIR 8 wk	0.0 (8)	0.0 (8)	0.0 (8)	0.0 (8)	0.0 (8)	0.0 (8)	0.0 (8)	0.0 (8)	0.0 (8)	0.0 (8)	0.0 (8)	0.0 (8)
HFWD+AIR 8 wk	0.0 (8)	0.0 (8)	0.0 (8)	0.0 (8)	0.0 (8)	0.0 (8)	0.0 (8)	0.0 (8)	0.0 (8)	0.0 (8)	0.0 (8)	0.0 (8)
STD+SIL 8 wk	4.0 (8)	2.0 (8)	4.0 (8)	2.0 (8)	4.0 (8)	2.0 (8)	4.0 (8)	1.0 (8)	0.0 (1) 3. 0 (7)	0.0 (1) 1. 0 (3) 2. 0 (3) 3. 0 (1)	0.0 (1) 5. 0 (7)	0.0 (1) 1. 0 (3) 2. 0 (4)
HFWD+SIL 8 wk	4.0 (8)	3.0 (8)	4.0 (8)	3.0 (8)	4.0 (8)	3.0 (8)	4.0 (8)	2.0 (8)	3.0 (8)	1. 0 (2) 2. 0 (4) 3. 0 (2)	0.0 (2) 5. 0 (6)	0.0 (2) 2. 0 (5) 3. 0 (1)

Semiquantitative histopathologic assessment was used to determine distribution and severity of silica-induced lesions in the lung at 0, 4 and 8 wk post-exposure to silica. Distribution: 0 = none, 1 = focal, 2 = locally extensive, 3 = multifocal, 4 = multifocal and coalescent, and 5 = diffuse. Severity/Area: 0 = none, 1 = minimal (<10%), 2 = mild (10–25%), 3 = moderate (26–50%), 4 = marked (51–75%), and 5 = severe (76–100%). n values for each score are indicated in parenthesis.

**Fig. 6:**
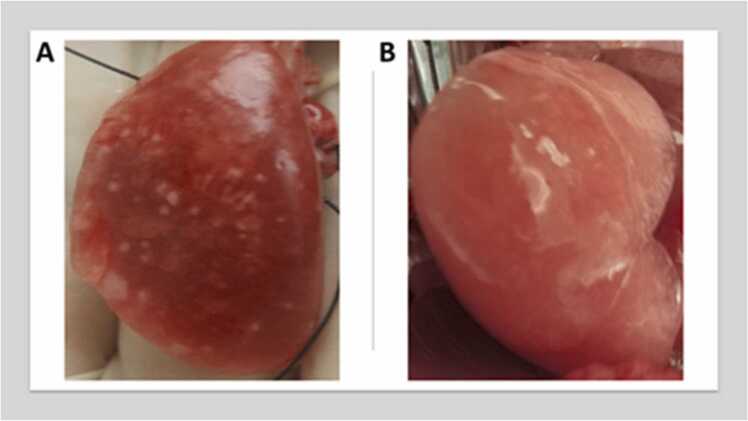
Images of lung from animals 8 wk after cessation of silica exposure. Red inflamed lung tissue and lesions were clearly visible in the left lung obtained from HFWD+SIL (A) compared to the STD+SIL (B) animals at 8 wk.

At 8 wk, lungs obtained from the HFWD+SIL cohort were visibly different from those of the STD+SIL cohort (Fig. 6, Suppl. Fig. 1). HFWD+SIL lungs were red, inflamed, and contained many visible white lesions on the surface, whereas the lungs from STD+SIL-treated animals did not. This observation is in agreement with the histological observation that at 8 wk HFWD+SIL had potentiated pulmonary inflammation compared to STD+SIL exposure. The HFWD+SIL 8 wk lungs were also comparable to images of three-month post-exposure lung images found in the study by Komai et al. (2019), in which rats were intratracheally instilled with 20 µg of crystalline silica to induce progressive massive fibrosis (PMF) [29].

## 4 Discussion

The aim of this study was to determine whether HFWD-consumption has the potential to alter silica-induced inflammatory responses in the lung. Our results indicate that HFWD consumption alters the histopathology of silica-induced lung injury and inflammation, revealing exacerbation of silica-induced lung inflammation and fibrosis in the F344 rat.

BAL total cell count and cell differentials were used to measure silica-induced pulmonary inflammation and BAL LDH was used as an indicator of lung cytotoxicity (Fig. 2). While STD+SIL exposure induced cytotoxicity at all timepoints, the increase in BAL LDH levels were sustained and did not increase over time. On the other hand, LDH levels were elevated in HFWD+SIL-exposed animals at 0 wk to a degree equal to the STD+SIL group, but at 4 and 8 wk, BAL LDH levels were significantly greater than that of the STD+SIL-exposed animals and was increased over time within the HFWD+SIL group. BAL total cell and differential cell counts indicated that a significantly greater influx of inflammatory cells into the lung had occurred in the HFWD+SIL-exposed animals compared to the STD+SIL-exposed cohort, and BAL cell numbers were increased over time within the HFWD+SIL group. Interestingly, the HFWD+SIL-induced BAL cytokines, TNF-α, KC/GRO, IL-1β, IL-5, IL-6, were increased in a “bell-shaped” profile over time, i.e., peaking at 4 wk and then declining, which was unique to this exposure group (Fig. 3). HFWD+SIL-induced BAL cytokine levels were not different from those of the STD+AIR control at 0 wk, but they then significantly increased at 4 wk, followed by a reduction at 8 wk. It is not understood why the various cytokines declined at 8 wk, while BAL total and differential cell counts continued to increase at 8 wk. The histopathological findings were consistent with the changes occurring in the BAL fluid, indicating that pulmonary inflammation continued to increase in distribution and severity from 4 to 8 wk in HFWD+SIL-treated animals, even more so than in the STD+SIL group (Fig. 4). We postulate that the lack of a strict agreement between the rising pulmonary inflammation and the decreased levels of BAL pro-inflammatory cytokines within the HFWD+SIL group at 8 wk is indicative of dysregulated and persistent inflammation in the lung of rats consuming a HFWD and exposed to silica, resulting in a greater severity of alveolar inflammation and fibrosis compared to the STD+SIL group.

Previously, we described the effects of HFWD consumption on systemic inflammation [25]. In that study, HFWD-consumption increased serum pro-inflammatory cytokines (IFN-γ, KC/GRO, TNF-α, IL-1β, IL-4, IL-5, IL-6, IL-10, IL-13) compared to STD+AIR-fed controls; however, there was no effect of the HFWD on blood cell count. In this pulmonary study, HFWD consumption increased chemotactic factor KC/GRO in the BAL at 4 and 8 wk, and interleukins IL-4 at 8 wk, IL-5 at 0, 4 and 8 wk, and IL-6 at 0 and 8 wk, compared to STD+AIR controls. HFWD-consumption also increased BAL total cells, lymphocytes, neutrophils, and macrophages at 4 wk compared to the STD+AIR control cohort. These results indicate that consumption of a HFWD not only induced systemic inflammation, as shown earlier [25], but that it also induced an inflammatory environment in the lung. We postulate that this HFWD-induced low-grade, systemic inflammation “primed” the lung and contributed to the exacerbated inflammatory response to silica dust inhalation.

In Thompson et al. [25], silica inhalation’s profound effects on serum leptin and adiponectin levels were described: a significant reduction in both adipokines at 8 wk followed silica inhalation, regardless of whether animals were fed a HFWD or STD diet [25]. In the present study we found that HFWD-consumption significantly increased silica-induced lung inflammation and fibrosis at 8 wk. We hypothesize that alteration in the levels of adipokines by silica dust and HFWD-consumption are compounding factors in the exacerbated effect of HFWD+SIL-induced lung injury. Adiponectin has a protective role against lung injury [30], [31] through its role of maintaining lung immune homeostasis [32], [33]. Previously [25], we found that, regardless of the diet consumed, silica-inhalation reduced both serum adiponectin and leptin at 8 wk in our animal model. Surprisingly, HFWD-consumption alone had lesser effects on adipokines in our animal model, which we attributed to the obesity-resistance of the F344 strain [34]. It is important to note, however, that the decrease in adipokines from 4 to 8 wk induced by silica-inhalation in the HFWD+SIL group was associated with a significant increase in the severity of lung inflammation and fibrosis from 4 to 8 wk, regardless of diet (Fig. 4, Fig. 5). Other studies confirm an adipokine connection to protection of the lung. Lean mice given leptin, prior to LPS intratracheal instillation, were protected from LPS-induced lung injury and corticosterone increase [35]. In a study of obese mice, investigators reported that decreased adiponectin was associated with increased levels of serum and lung selectins (proteins for WBC adherence to endothelial cells) and decreased lung adherins (cell adhesion proteins); when pre-treated with adiponectin, susceptibility to LPS-induced lung injury was reversed [36]. We speculate that altered serum adipokines, altered by combination of HFWD-consumption and silica-induced effects, played a role in the exacerbation of silica-induced injury.

The findings made in the present investigation are reminiscent of the World Trade Center first responder studies, in which the presence of metabolic syndrome biomarkers was associated with increased susceptibility to severe lung injury from dust inhalation. Our findings may be relevant to other investigations of the effects of HFWD-consumption-induced obesity on responses to other occupational particle exposures, including mixed occupational exposures. Finally, the approach used in this study would be useful for risk assessment and the prevention of occupational lung disease.

### 4.1 Limitations and future directions

NLRP3 inflammasome activation, an important mechanism in the pathogenesis of silicosis, is activated by crystalline silica both in alveolar macrophages (AM) and airway epithelial cells [14], [37]. We measured BAL IL-1β as a marker of NLRP3 activation; however, measurement of additional NLRP3 endpoints, such as the expression of NLRP3 proteins, IL-18, and caspase-1, would provide further insight into mechanistic changes in inflammasome activation resulting from the synergistic interaction between HFWD-consumption and silica exposure in the lung. While our rodent model shares many similarities with silicosis in humans, there are also distinct differences in the effects of the dust in the two species. Similarities include accumulation of silica particles in the same regions of the lung; phagocytosis of particles by AM in alveoli and respiratory bronchioles [38]; alveolar lipoproteinosis [13], [39], [40]; progression of silicosis after cessation of exposure [13], [38], [41]; fibrosis of lymph nodes or lymphoid tissue [40]; and pulmonary inflammation and interstitial fibrosis [21], [23]. Distinct differences between our rodent model and human silicosis include the abundance of granulomas in the rat lung, in comparison to the human disease in which silica nodules tend to be fibrotic with onion-like layers surrounding the silica particle core [42] and the absence in rats of progressive massive fibrosis, the most fatal form of silicosis in humans. Finally, our study used “aged” silica, not freshly fractured silica to which workers are exposed and which causes an even greater pulmonary inflammatory response than aged silica [43].

## 5 Summary and conclusions

We conclude that HFWD-consumption exacerbates silica-induced pulmonary inflammation and fibrosis in the F344 rat model of silicosis. HFWD-consumption induces serum pro-inflammatory cytokines [25], [44] and reduces adiponectin [25], [45], and in combination, may prime the lung for an elevated inflammatory response following silica inhalation. While evidence of controlled but progressive pulmonary inflammation leading to fibrosis over time was obtained in STD+SIL-exposed rats, it appears that these inflammatory responses are dysregulated in the HFWD+SIL exposure groups. This dysregulation results in steadily increasing inflammatory cell infiltration into the lung, greater increases in BAL pro-inflammatory cytokine levels, and intensification of pulmonary inflammation and progression of fibrosis. Our results suggest that an increased susceptibility to silica-induced lung injury may occur in workers who consume a HFWD.

## CRediT authorship contribution statement

**Janet A. Thompson:** Conceptualization, Methodology, Data curation, Visualization, Writing – original draft, Supervision. **Richard A. Johnston:** Methodology, Investigation. **Roger E. Price**: Conceptualization, Methodology. **Ann F. Hubbs:** Conceptualization, Methodology, Investigation. **Michael L. Kashon:** Data curation. **Walter McKinney:** Conceptualization, Methodology, Investigation. **Jeffrey S. Fedan:** Conceptualization, Methodology, Data curation, Visualization, Investigation, Supervision, Writing – review & editing.

## Declaration of Competing Interest

The authors declare that they have no conflicts of interest in relation to this publication.

## Data Availability

The original data are available at https://www.cdc.gov/niosh/data/datasets/RD-1032-2022-0/.
