# Supplementary figures and images for: High-fat Western diet consumption exacerbates silica-induced pulmonary inflammation and fibrosis
Source: Toxicol Rep. 2022 May 2;9:1045–53. doi: 10.1016/j.toxrep.2022.04.028 (PMC9350629; doi:10.1016/j.toxrep.2022.04.028)

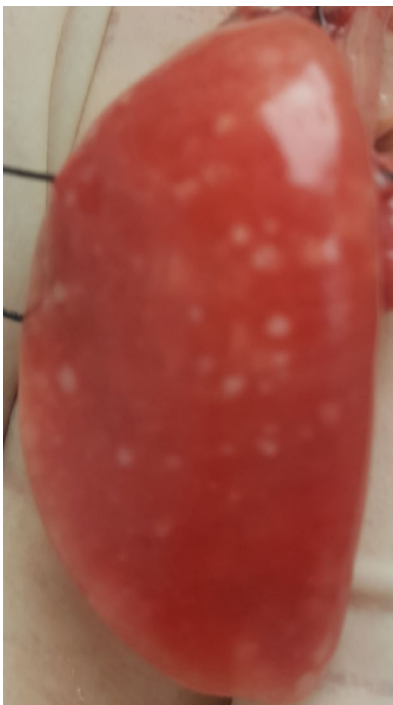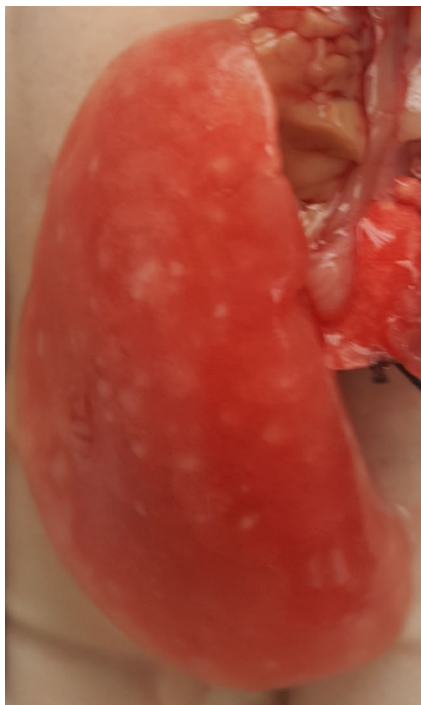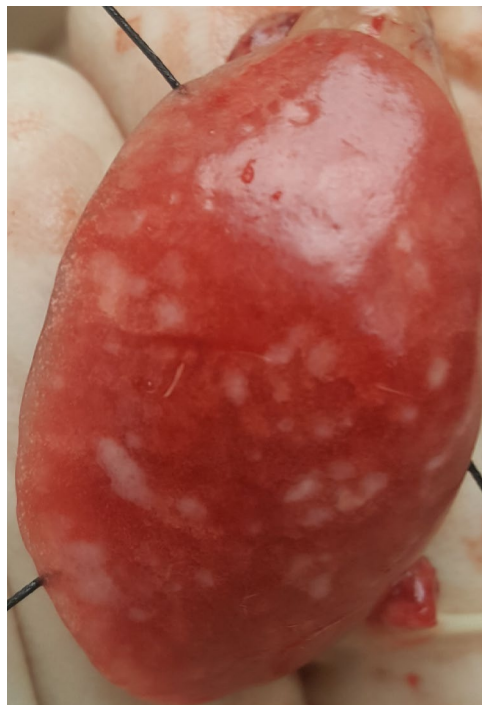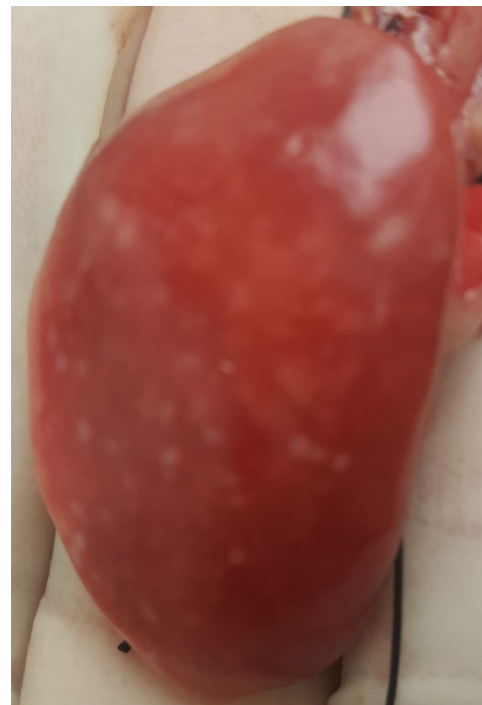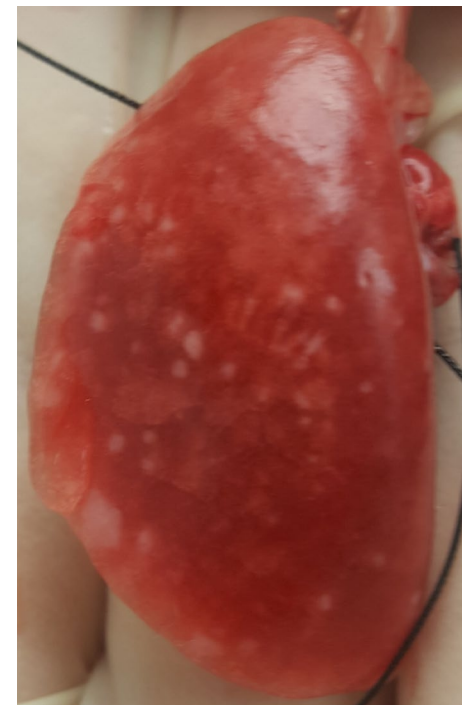

Supplement: Supplementary material [file mmc1.pdf]
