# Supplementary material for: High-fat Western diet consumption exacerbates silica-induced pulmonary inflammation and fibrosis
Source: Toxicol Rep. 2022 May 2;9:1045–53. doi: 10.1016/j.toxrep.2022.04.028 (PMC9350629; doi:10.1016/j.toxrep.2022.04.028)
Supplement: Supplementary material [file mmc2.docx]

Supplemental Fig. 1. Images of inflated lungs from animals fed a HFWD and exposed to silica at 8 wk post exposure. Red inflamed lung tissue and white lesions were clearly visible in lungs of animals from this exposure group, as also shown in Fig. 6. n = 5.
